# Carpal Tunnel Syndrome with Paracoccidioidomycosis

**DOI:** 10.3201/eid1808.120153

**Published:** 2012-08

**Authors:** Felipe von Glehn, Alfredo Damasceno, Noelle Miotto, Estephania P. Naseri, Lilian T.L. Costallat, Marcondes C. França, Anamarli Nucci, Marcelo C. Ramos

**Affiliations:** University of Campinas, São Paulo, Brazil

**Keywords:** Carpal tunnel syndrome, Paracoccidioidomycosis, fungal infection, arthritis, immunosuppressive therapy, fungi, letter

**To the Editor**: Paracoccidioidomycosis, a systemic mycosis caused by *Paracoccidioides brasiliensis*, is endemic to rural areas of Latin America (*1*). Persons are infected early in life by inhaling the fungus propagules, which reach the lower airway and cause primary complex (*2*). The most common clinical manifestation of paracoccidioidomycosis, which occurs with the chronic multifocal form, is characterized by pulmonary and extrapulmonary (e.g., skin, central nervous system, osteoarticular system) involvement, which occurs after a prolonged latency period (*2*). Carpal tunnel syndrome (CTS) is seldom associated with pyogenic agents (*3*), *Mycobacterium tuberculosis* (*4*), or fungal agents (*5*). Few reports have described paracoccidioidomycosis in immunosuppressed patients (*6*). We report a rare case of flexor tenosynovitis and severe CTS in the context of reactivated, chronic paracoccidioidomycosis infection.

A 63-year-old white male agricultural worker from São Paulo, Brazil, reported insidious and progressive pain, numbness, and tingling in his right hand and fingers, which began in April 2009. His medical history included symmetric polyarthritis of hands, ankles, and knees, which had been diagnosed elsewhere as seronegative rheumatoid arthritis in 2006. At that point, he also had chronic cough; a computed tomographic (CT) scan of the chest showed small nodules and mild interstitial fibrosis, and sputum specimens were negative for fungi or mycobacteria by microscopy. For treatment, he received prednisone, leflunomide, meloxicam, and methotrexate. Hydroxychloroquine was added in March 2010 because of worsening polyarthritis. Pain in the right hand also increased, and infiltrations of the right carpal tunnel with methylprednisolone and lidocaine were performed in September and October 2010, with poor response. After that, physical examination showed mild edema and warmth of the flexor surface of the hand and reduced wrist motion. Phalen test and Tinel signs were positive. In February 2011, an outpatient electrophysiologic evaluation showed a severe right focal demyelination of the median nerve at the wrist and mild acute denervation in the abductor pollicis brevis muscle, consistent with CTS.

In August 2011, the patient was admitted to the hospital of the University of Campinas, São Paulo, Brazil, with poor general health, fever, cutaneous nodules on the trunk and limbs, and dyspnea. Purulent material drained from 2 fistulous nodules in the right thumb and forearm (Figure, panel A). A new CT scan of the chest indicated cystic bronchiectasis, bronchial wall thickening, and adjacent areas of consolidation. Microscopic examination of thumb secretion and sputum samples by using 10% potassium hydroxide revealed the characteristic pilot’s wheel appearance of *Paracoccidioides brasiliensis,* showing multiple-budding yeast cells with well-defined refringent double walls (*7*) (Figure, panel C). Grocott-Gomori stain of a skin biopsy specimen demonstrated yeast with the same microscopic features (*7*). Serologic tests for *Paracoccidioides* spp. were negative. Blood and thumb secretion cultures were negative for *Mycobacterium* spp. and fungi. Magnetic resonance imaging of the right wrist and forearm showed diffuse inflammatory infiltrates with signs of tenosynovitis and fluid collection involving the flexor compartment and extending to areas corresponding to fistulous skin lesions (Technical Appendix).

**Figure:**
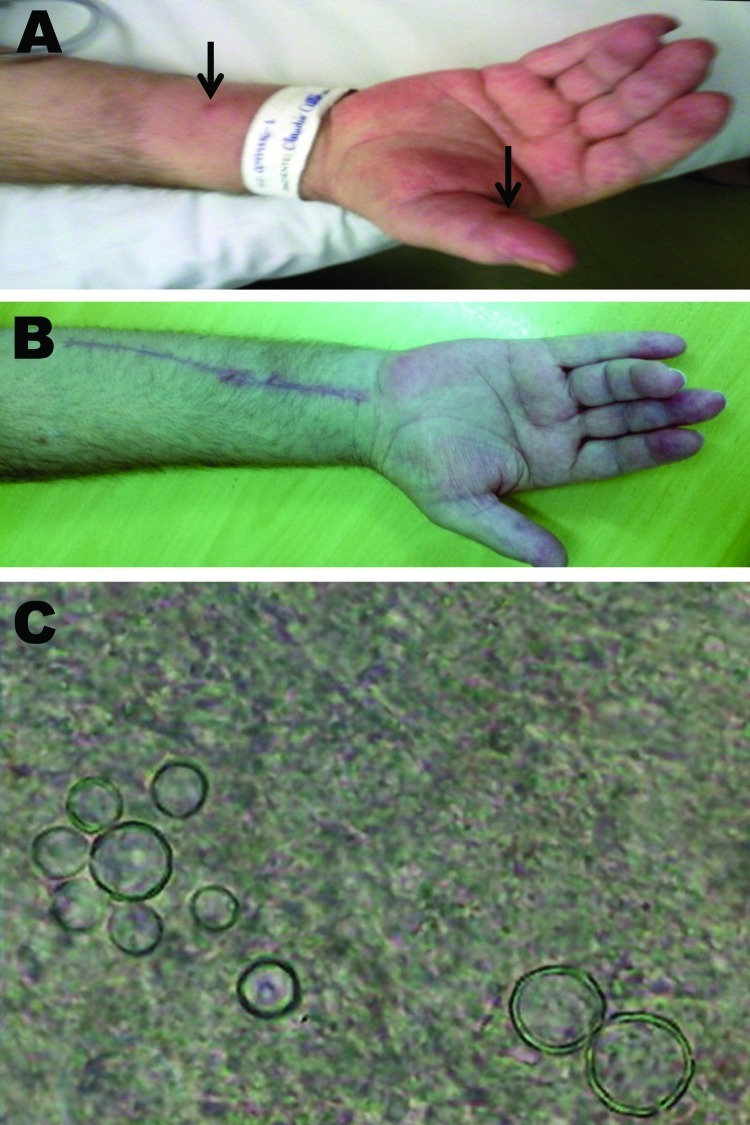
A) Edema and erythema of the flexor surface of the hand of patient with paracoccidioidomycosis, carpal tunnel syndrome, and flexor tenosynovitis, Brazil. Note a fistulous pustulous nodule in the right thumb and forearm (arrows) and flexor contracture of the fourth finger. B) Flexor surface of the hand and forearm after surgery. C) *Paracoccidioides brasiliensis* was directly identified on the thumb secretion, sputum, and flexor tenosynovectomy specimen by using a 10% potassium hydroxide preparation. This image was obtained from the thumb secretion. Note the characteristic multiple-budding yeast cells (pilot's wheel) with the well-defined refringent double wall.

Intravenous co-trimoxazole was prescribed, followed by oral itraconazole. Immunosuppressant drugs were withdrawn. After the patient’s general health stabilized, he underwent open carpal tunnel release, flexor tenosynovectomy, and collection of the purulent drainage. When evaluated 5 months after hospital discharge, his right hand symptoms and polyarthritis had almost completely resolved (Figure, panel B). A neurophysiologic examination demonstrated a mild improvement in distal median neuropathy. Results of serologic assessment for rheumatoid factor and antibodies against cyclic-citrullinated peptide were negative. Also, no signs of bone erosions or subcortical cysts were shown on radiograph of wrist and hand joints, which does not support the diagnosis of seronegative rheumatoid arthritis.

Although a direct search for fungi and mycobacterial agents was initially negative, paracoccidioidomycosis should still have been included in the differential diagnosis for this patient, who exhibited arthritis and pulmonary symptoms and had the risk factors of heavy smoking and living in a paracoccidioidomycosis-endemic region. The initial chest CT scan did not rule out paracoccidioidomycosis (*7*). However, seronegative rheumatoid arthritis was diagnosed and treated. When the patient arrived in our hospital, systemic manifestations, severe pulmonary compromise, and CTS of the right hand were the main features of his condition, and *P. brasiliensis* was detected on direct microscopic observation of sputum and thumb secretions. The central nervous system is a frequent extrapulmonary site of damage by paracoccidioidomycosis (*2**,**8**,**9*), but for paracoccidioidomycosis to cause CTS is unusual. The patient received immunosuppressive drugs during a 5-year period. The immunosuppressive treatment could contribute to reactivation of pulmonary quiescent infection foci and hematogenous fungal spread. Infiltrations of the wrist with corticosteroids could facilitate and enhance local fungal proliferation after hematogenous dissemination. Factors such as inoculum size, pathogenicity and strain virulence, and patient’s immune status could explain the development and severity of disease (*2*). Immunocompromised patients, particularly those with cell-mediated immune impairment, are at greatest risk for severe disseminated paracoccidioidomycosis (*6*), as occurred in this patient. Identifying antibodies against *Paracoccidioides* spp. in patient’s serum would have helped monitor the host response to treatment (*2*), but he was seronegative, probably because of his immunosuppressed state. Paracoccidioidomycosis serologic testing would be useful early in the disease to help distinguish between seronegative rheumatoid arthritis and reactive arthritis. Paracoccidioidomycosis should always be suspected in *P. brasiliensis*–endemic areas.

Technical Appendix Magnetic resonance image of the right wrist and forearm of patient with paracoccidioidomycosis, Brazil.
