# Supplementary material for: Carpal Tunnel Syndrome with Paracoccidioidomycosis
Source: Emerg Infect Dis. 2012 Aug;18(8):1390–2. doi: 10.3201/eid1808.120153 (PMC3414040; doi:10.3201/eid1808.120153)
Supplement: Technical Appendix — Magnetic resonance image of the right wrist and forearm of patient with paracoccidioidomycosis, Brazil. [file 12-0153_Techapp-s1.pdf]

Article DOI: http://dx.doi.org/10.3201/eid1808.120153

Carpal Tunnel Syndrome with Paracoccidioidomycosis

Technical Appendix

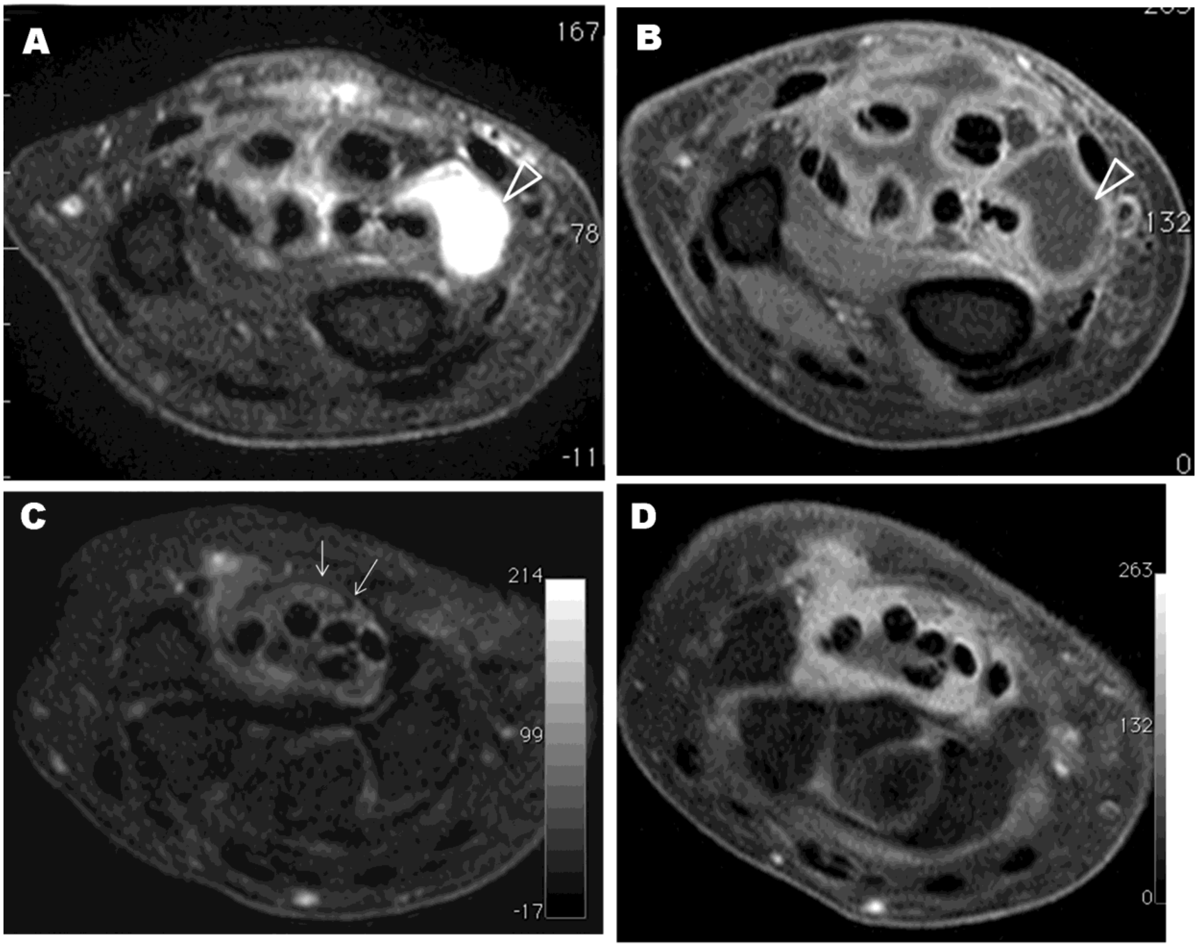

Technical Appendix Figure. Magnetic resonance image of the right wrist and forearm of patient with paracoccidioidomycosis, Brazil. Diffuse inflammatory infiltrates are shown with signs of tenosynovitis and fluid collections involving the flexor compartment and extending to areas corresponding to fistulous skin lesions. Axial T2-weighted, fat-saturated image showing increased signal intensity areas of the flexor compartment, with arrowhead indicating fluid collection (A); after gadolinium administration showing diffuse contrast enhancement (B) (arrowhead indicates fluid collection);increased signal intensity of the flexor compartment, including the median nerve, with arrows showing bulging of the flexor retinaculum (C); and after gadolinium administration showing diffuse contrast enhancement extending to subcutaneous tissue (D).
